# Supplementary material for: The Induction of Long-Term Potentiation by Medial Septum Activation under Urethane Anesthesia Can Alter Gene Expression in the Hippocampus
Source: Int J Mol Sci. 2023 Aug 19;24(16):12970. doi: 10.3390/ijms241612970 (PMC10454684; doi:10.3390/ijms241612970)
Supplement: Supplementary file 1 [file ijms-24-12970-s001.zip › Supplementaries.pdf]

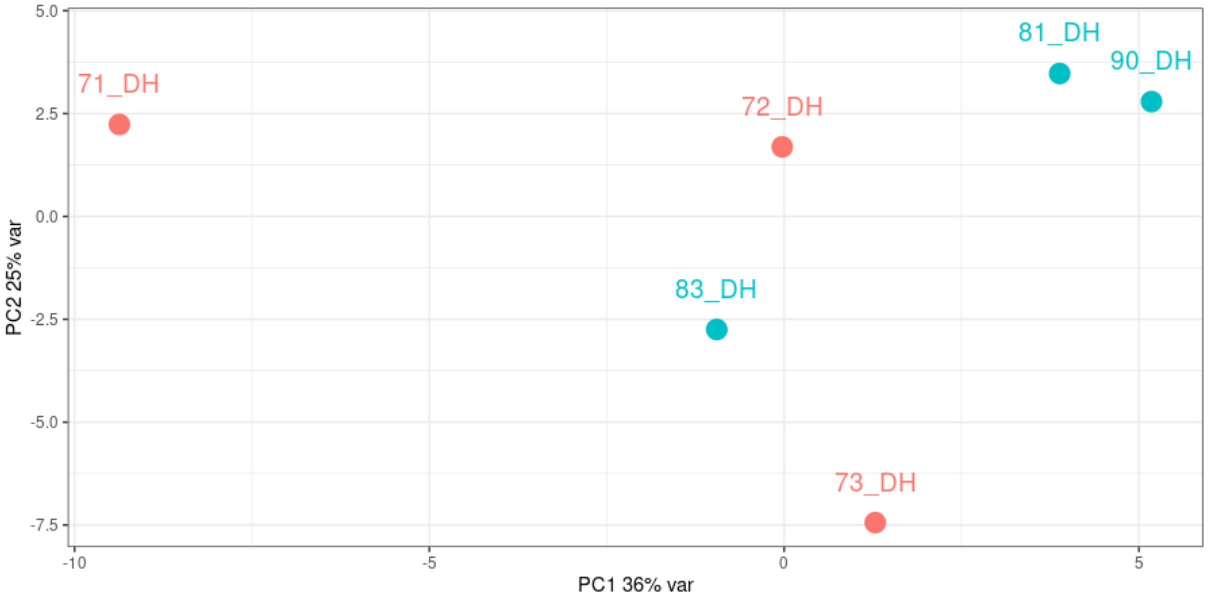

Supplementary Figure S1. Principle component analysis score plot. Control samples are shown in red; samples with cholinergic deficit are shown in blue.

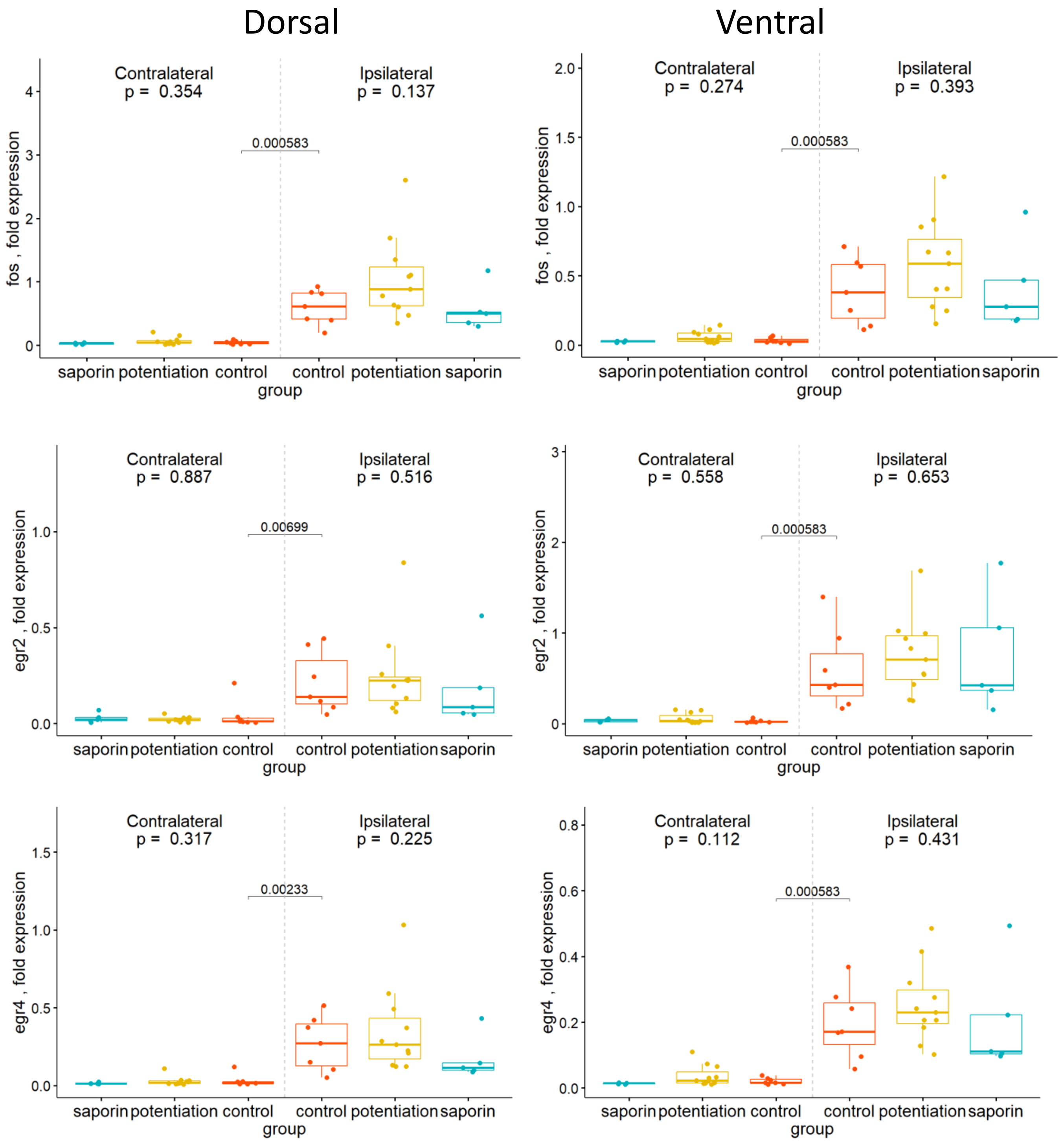

Supplementary Figure S2. Changes in the expression of bdnf and ngf genes in dorsal and ventral parts (DH and VH, respectively) of the left (contralateral) and right (damaged by implanted electrode) hippocampi after high frequency stimulation of the dorsal medium septal area in the control animals (potentiation) and animals with degeneration of cholinergic neurons in the medial septal area (saporin). *p*-values are shown for Kruskal–Wallis test for comparison of three groups in one hemisphere. Interhemispheric comparisons were performed only in the control group using the Mann–Whitney test. For the intergroup comparisons, p.adjusted was calculated using Benjamini-Hochberg method. Differences were considered as significant at p.adjusted < 0.05. For interhemispheric comparisons, exact p-values are shown.

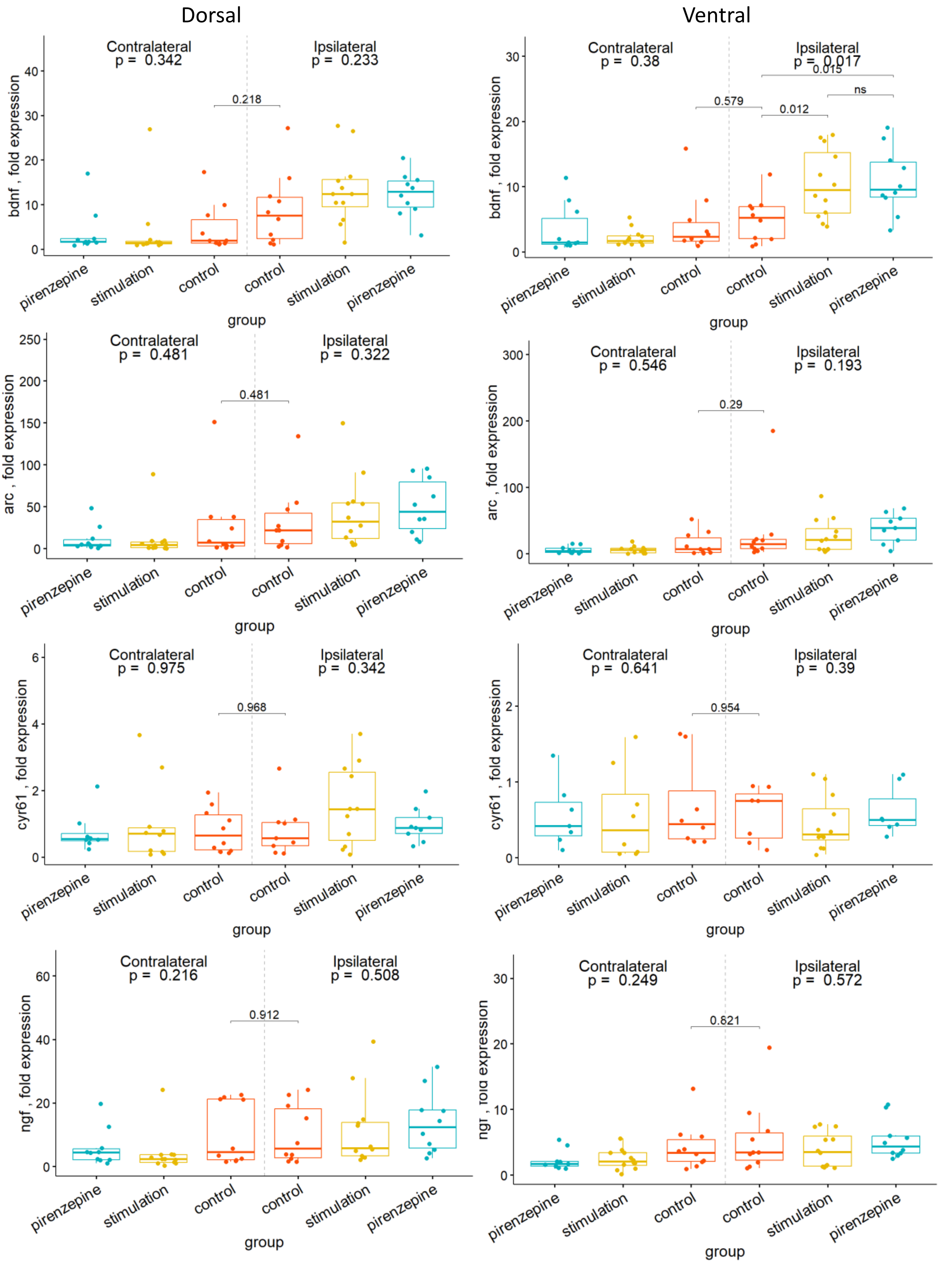

Supplementary Figure S3. Changes in the expression of early genes in dorsal and ventral parts (DH and VH, respectively) of the left (contralateral) and right (damaged by implanted electrode) hippocampi after rhythmic low frequency stimulation of the ventral hippocampal commissure in the control animals (stimulation) and animals injected with pirenzepine (pirenzepine). *p*-values are shown for Kruskal–Wallis test for comparison of three groups in one hemisphere. Interhemispheric comparisons were performed only in the control group using the Mann–Whitney test. For the intergroup comparisons, p.adjusted was calculated using Benjamini-Hochberg method. Differences were considered as significant at p.adjusted < 0.05. For interhemispheric comparisons, exact p-values are shown.

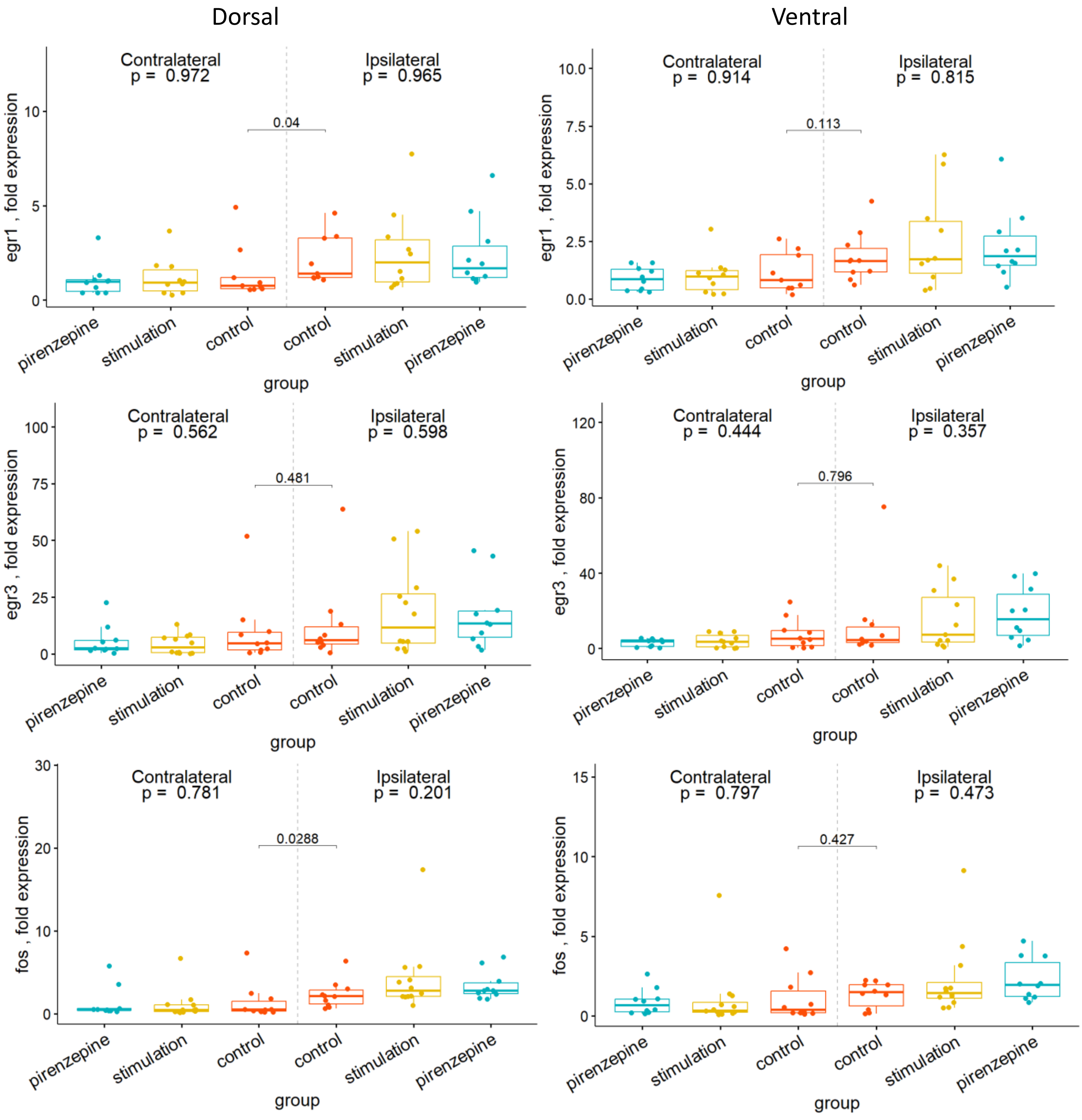

Supplementary Figure S4. Changes in the expression of early genes in dorsal and ventral parts (DH and VH, respectively) of the left (contralateral) and right (damaged by implanted electrode) hippocampi after rhythmic low frequency stimulation of the ventral hippocampal commissure in the control animals (stimulation) and animals injected with pirenzepine (pirenzepine). *p*-values are shown for Kruskal–Wallis test for comparison of three groups in one hemisphere. Interhemispheric comparisons were performed only in the control group using the Mann–Whitney test. For the intergroup comparisons, p.adjusted was calculated using Benjamini-Hochberg method. Differences were considered as significant at p.adjusted < 0.05. For interhemispheric comparisons, exact p-values are shown.

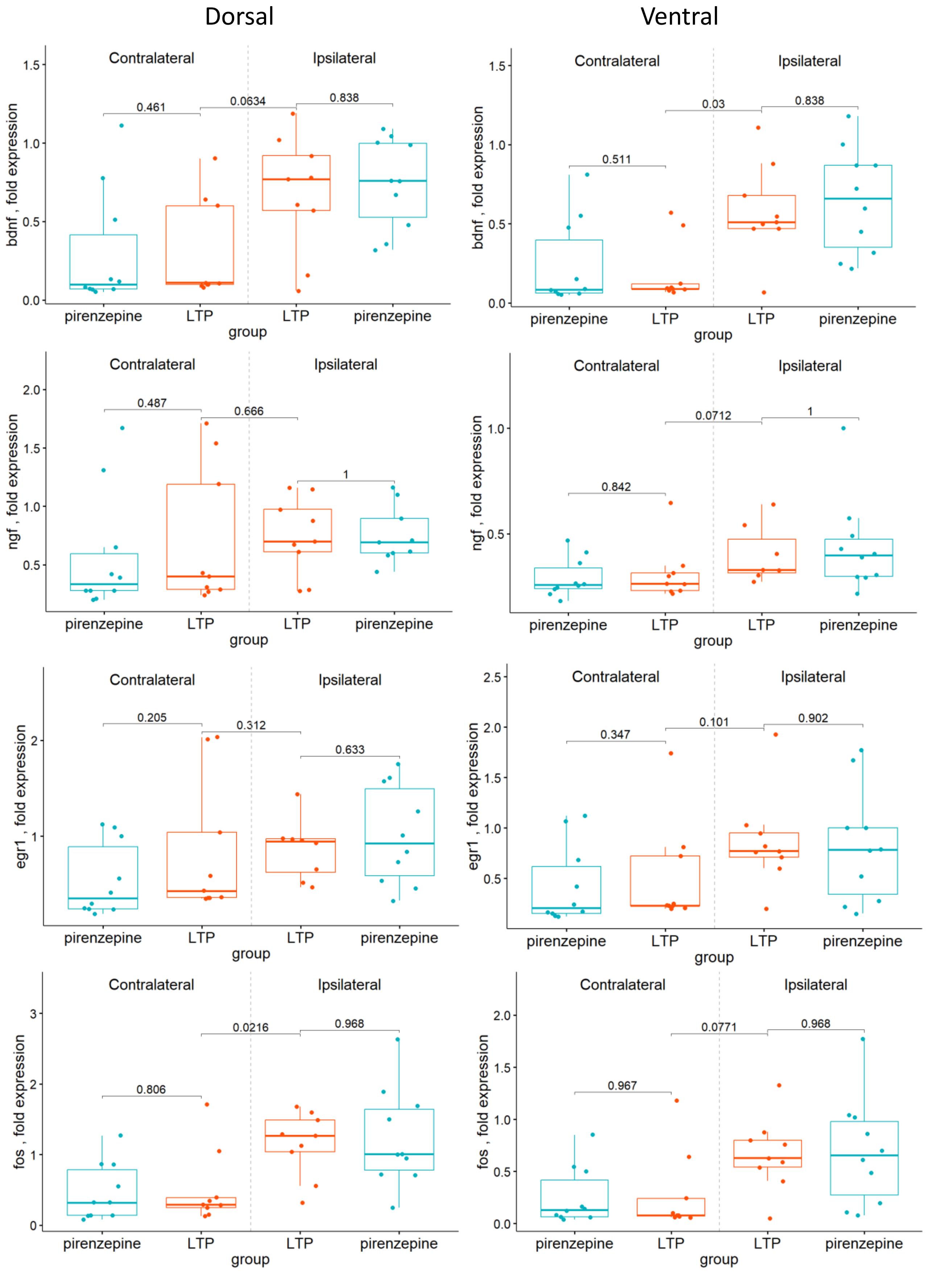

Supplementary Figure S5. Changes in the expression of early genes in dorsal and ventral parts (DH and VH, respectively) of the left (contralateral) and right (damaged by implanted electrode) hippocampi after high frequency stimulation of the dorsal medium septal area in the animals (LTP) intracerebroventricularly treated with saline (LTP) or pirenzepine (pirenzepine). *p*-values are shown for Mann-Whitney test for comparison in one hemisphere.
